# The Role of Social Contacts and Original Antigenic Sin in Shaping the Age Pattern of Immunity to Seasonal Influenza

**DOI:** 10.1371/journal.pcbi.1002741

**Published:** 2012-10-25

**Authors:** Adam J. Kucharski, Julia R. Gog

**Affiliations:** Department of Applied Mathematics and Theoretical Physics, University of Cambridge, Cambridge, United Kingdom; Emory University, United States of America

## Abstract

Recent serological studies of seasonal influenza A in humans suggest a striking characteristic profile of immunity against age, which holds across different countries and against different subtypes of influenza. For both H1N1 and H3N2, the proportion of the population seropositive to recently circulated strains peaks in school-age children, reaches a minimum between ages 35–65, then rises again in the older ages. This pattern is little understood. Variable mixing between different age classes can have a profound effect on disease dynamics, and is hence the obvious candidate explanation for the profile, but using a mathematical model of multiple influenza strains, we see that age dependent transmission based on mixing data from social contact surveys cannot on its own explain the observed pattern. Instead, the number of seropositive individuals in a population may be a consequence of ‘original antigenic sin’; if the first infection of a lifetime dominates subsequent immune responses, we demonstrate that it is possible to reproduce the observed relationship between age and seroprevalence. We propose a candidate mechanism for this relationship, by which original antigenic sin, along with antigenic drift and vaccination, results in the age profile of immunity seen in empirical studies.

## Author Summary

The way in which a population builds immunity to influenza affects outbreak size and the emergence of new strains. However, although age-specific immunity has been widely discussed for the 2009 influenza pandemic, the age profile of immunity to seasonal influenza remains little understood. In contrast to many infections, the proportion of people immune to recent strains peaks in school-age children then reaches a minimum between ages 35–65, before rising again in older age groups. Our results suggest that rather than variable mixing between different age groups being solely responsible, the pattern may be shaped by an effect known as ‘original antigenic sin’, by which the first infection of a lifetime dictates subsequent immune responses: instead of developing antibodies to every new virus that is encountered, the immune system may reuse the response to a similar virus it has already seen. The framework we describe, which extends theoretical models to allow for comparison with data, also opens the possibility of investigating the mechanisms behind patterns of immunity to other evolving pathogens.

## Introduction

Influenza A evolves over time, escaping the immunity of human host populations [1]. As a result, individuals are exposed to a range of different strains over a lifetime, and different age groups have varying levels of antibodies to particular strains, depending on which viruses they have seen. Several serological studies during the 2009 influenza pandemic also considered recent seasonal H1N1 and H3N2 strains, with haemagglutination-inhibition (HI) titres given for different age groups. Across a number of countries, the data all follow a distinct pattern [2], [3], [4], [5], [6], [7], [8]: a high proportion of individuals are seropositive (HI titre>40) in adolescence, followed by a clear decrease in seropositivity between adolescence and age 60–65, before a rise in the older ages.

Heterogeneity between age groups has been much studied in an epidemiological context [9], [10], and recent work used serological data for varicella and parvovirus to infer transmission rates between age groups [11]. However, despite the increasingly availability of social contact data [12], [13], it has previously been difficult to compare mathematical model outputs with data from serological studies for seasonal influenza: the proliferation of variables required as the number of strains in the model increases makes it technically challenging to look at the long term impact of different assumptions.

Progress has recently been made by introducing age structure to a multi-strain model, allowing the effect of influenza dynamics on population immunity to be examined in more detail [14]. Here, an extended version of this model is used to examine the possible causes of the unusual age distribution of seropositivity to seasonal influenza A in humans. A number of candidate factors are included: basic reproductive ratio (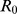); heterogeneous mixing between age classes; cross-immunity between strains; vaccination effectiveness. We also consider ‘original antigenic sin’ (OAS) [15], a theory that suggests that previous infection dominates subsequent immune responses: rather than develop antibodies to every new epitope that is encountered, if strains are antigenically similar, the immune system may reuse antibodies previously raised against the epitopes of an old strain, instead of developing immunity to the novel ones.

A simulation-based maximum-likelihood analysis is used to quantitatively compare the consequences of different assumptions with serological data from Australia [2] and Finland [3]. The data are compared with the degree of immunity calculated in the model by imposing the assumption that individuals with HI titre >40 are immune, and do not transmit infection. Although the precise relationship between the two is unclear, it has previously been shown that an HI titre >40 correlates with protection [16], [17].

This framework is used to assess the contribution of the different candidate factors. In particular, we see that if mixing follows physical interactions seen in social contact data and OAS is included in the model, it is possible to recreate the patterns seen in these serological studies.

## Methods

### Data

We selected two serological studies that tested age cohorts at a detailed resolution, with samples taken from specimens submitted for diagnostic testing in Finland in 2004/5 [3] and Australia after the first pandemic wave in 2009 [2]. Both studies tested for an HI titre >40, defined as seropositive, against seasonal H1N1 and H3N2 strains. As well as testing a range of subtypes and strains in a number of age groups, these studies took place in populations with similar demography and vaccination programmes.

### Mathematical Model

We use a seasonal model of influenza [18], in which the processes of disease transmission and antigenic evolution are separated by considering each annual epidemic individually, with mutation occurring between seasons. We assume that a single influenza strain circulates during each epidemic, and epidemics do not overlap. This appears to be a reasonable model for temperate regions, which are annually ‘seeded’ with influenza after low levels of prevalence over the summer [19], [20], [21], and which have low diversity of strains during epidemics [22], [23]. Our simulated sequence of epidemics begins in 1968 for H3N2 strains, and 1977 for H1N1, up to the HI assay test year (assumed to be 2005 and 2009 for Finland and Australia respectively). It is assumed that there is no interaction between influenza subtypes, and that both H1N1 and H3N2 circulate each year.

We define cross-immunity between strains in one of two ways. The first assumes that a fixed amount of antigenic change occurs each year. Strains are numbered by the year in which they appeared, increasingly sequentially, with cross-immunity decaying exponentially between years [3], [8]. We define 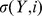 to be the probability an individual will transmit a challenge strain 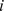, given previous exposure to strains in a set of strains 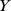, and assume that the immune response is dictated by the strain in 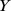 most antigenically similar to the new strain. Hence for strains that circulated 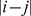 years apart,
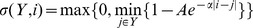
(1)where 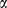 and 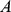 are parameters to be fitted.

The second definition of cross-immunity is based on the observation that large antigenic changes happens every few years [24], [25], [26]. Rather than strains changing each year, we assume that strains are collected into clusters that are of temporal size similar to those that actually circulated (Table S1). We assume that strains give partial cross-immunity to other strains in the same cluster, and cross-immunity decays exponentially between clusters. Hence Equation 1 remains the same, but 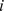 and 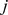 now index the cluster number instead of strain year. 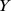 denotes the set of clusters previously seen, and the decay in cross-immunity is dictated by the distance between clusters, 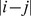.

It is assumed that original antigenic sin is generated by the first infection of a lifetime [14]. Suppose 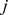 is the first strain (or cluster) seen, and the individual is subsequently exposed to strain (or cluster) 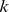. We assume that if 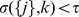, immunity to 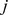 prevents the gain of any new immunity as a result of exposure to 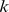. The parameter 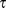 can be thought of as the ‘reach’ of OAS: if 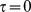, every infection will result in new immunity; if 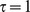, any degree of cross-immunity will lead to the existing response being reused.

### Age-dependent Mixing

To incorporate age-dependent mixing, we assume four age classes: infants (0–4); school children (5–14); younger adults (15–49); older adults (50–99). Age-dependent mixing is derived from the European POLYMOD survey in Finland [13], which includes data on both physical and conversational contacts, and previous theoretical results [14], [27] are extended to calculate the proportion of individuals that have been infected in each season (details given in Text S1). At the start of a new season, these values are used to calculate the proportion of the population who are aged 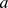 and would have the potential to transmit if they acquired infection. We denote this value by 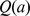, which can also be thought of the proportion who have no cross-immunity to the current strain.

### Demography and Vaccination

In Australia and Finland, the equilibrium age distribution of the population, 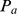, is relatively flat up to age 60, then decreases approximately linearly, reaching zero at around age 100 [28], [29]. We therefore use a simple piecewise age distribution in the model (Figure S1), with births, deaths and ageing occurring between each annual epidemic. Influenza vaccines are routinely offered to individuals over 65; we assume coverage is 50% in Finland [30] and 60% in Australia [31] and that vaccination effectiveness is 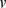. This is implemented by reducing 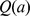 by a factor 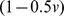 or 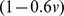 for 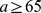 at the start of each season.

### Model Fitting

We assume that an HI titre >40 is protective. Let 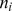 be number of individuals tested and 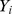 be number of seropositive individuals in each age cohort. The parameter set we fit is
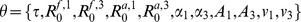
with definitions, and prior assumptions, given in Table 1. Given a set of parameters, the model prediction for immunity in age cohort 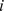 – the probability that a person sampled uniformly from that cohort will not transmit disease upon infection – is given by
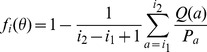
(2)where 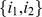 denote the age boundaries of cohort 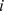. We can therefore calculate the likelihood of observing 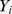 seropositive individuals in a sample of size 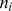 using a binomial probability mass function,
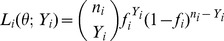
(3)which gives a log-likelihood of
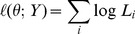
(4)Parameter inference is done via the Metropolis-Hastings algorithm, a Markov chain Monte Carlo method [32].

**Table 1:** Parameters fitted.

Parameter	Description	Prior
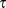	reach of OAS	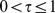
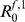	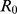 of subtype H1N1 in Finland	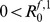
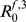	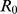 of subtype H3N2 in Finland	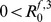
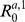	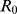 of subtype H1N1 in Australia	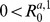
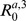	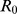 of subtype H3N2 in Australia	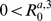
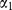	cross-immunity decay, 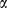 , for H1N1	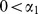
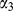	cross-immunity decay, 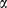 , for H3N2	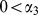
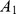	cross-immunity parameter for H1N1	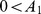
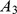	cross-immunity parameter for H3N2	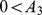
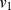	vaccination effectiveness against H1N1	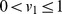
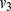	vaccination effectiveness against H3N2	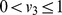

## Results

**Figure 1:**
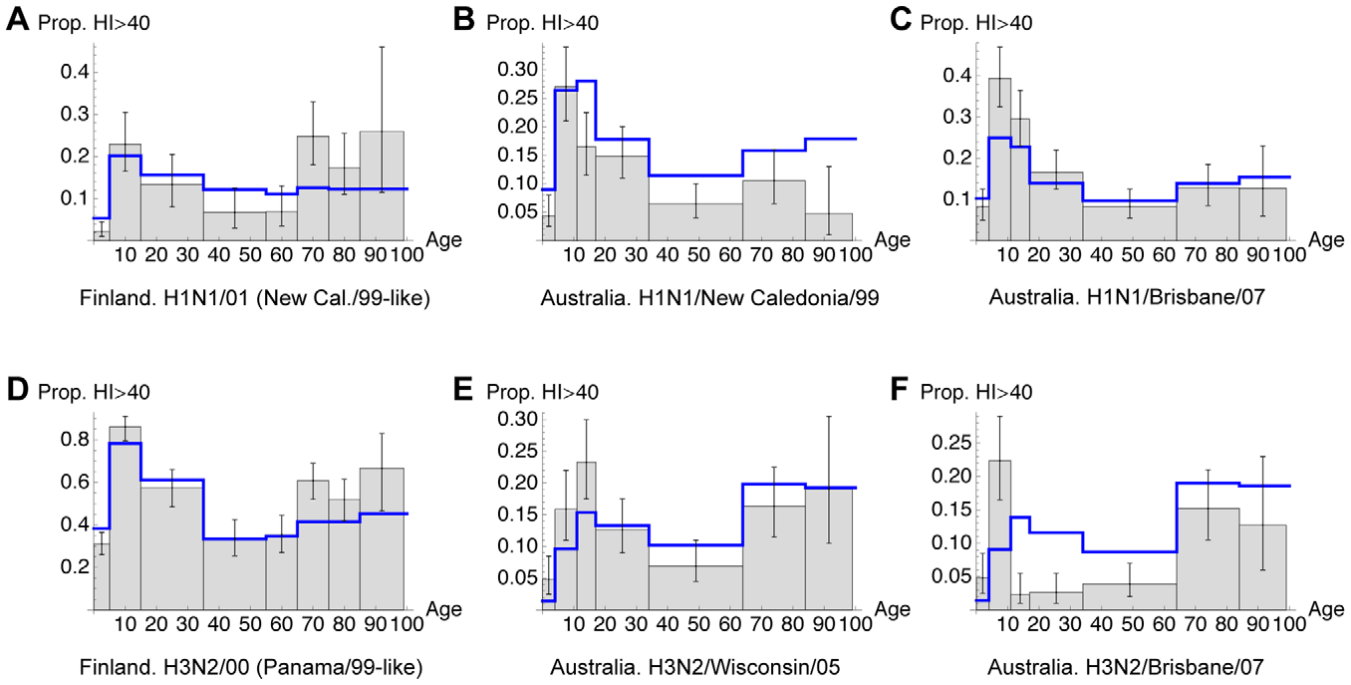
Comparison of model to data for Australia [**2**] and Finland [**3**]. Grey bars indicate observed seropositivity (proportion with HI titre >40) in each age cohort, with binomial confidence intervals given by black error bars. Blue lines show the age profile of immunity predicted by the best fitting model. The test year is 2005 for Finland and 2009 for Australia. A, proportion seropositive to H1N1/01 (New Cal./99-like strain) in Finland; B, H1N1/New Caledonia/99 in Australia; C, H1N1/Brisbane/07 in Australia; D, H3N2/00 (Panama/99-like) in Finland; E, H3N2/Wisconsin/05 in Australia; F, H3N2/Brisbane/07 in Australia.

Using this framework, we tested combinations of three different assumptions: 1) transmission derived from physical contacts or conversational contacts in the POLYMOD survey [13]; 2) clusters of strains or fixed amount of antigenic change each year; 3) OAS or no OAS. The eight possible models were compared using the AIC, corrected to avoid overfitting [33]. Table 2 shows that the model with physical contacts, OAS and clusters gave the best fit according to the AIC. In addition, the values of 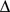, which give the difference in AIC compared to the best fitting model, suggest that models with transmission based on conversational contact data [13] all have less support under the AIC than those based on physical contacts. Figure 1 compares the six sets of data with the maximum likelihood fit of model 1, which best explained the data. The model captures the general shape of five of the data sets, in particular the drop in seropositivity after adolescence, but does not fit as well to the Australian data for H3N2/Brisbane/07 (Figure 1F); the substantial drop in immunity after childhood does not occur in the model.

**Table 2:** Comparison of different models.

Model	Mixing	Antigenic change	OAS	Parameters	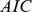	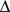
1	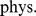 *	clusters	yes	11	505.9	0
2	phys.	clusters	no	10	515.1	9.2
3	phys.	yearly	yes	11	535.5	29.6
4	phys.	yearly	no	10	533.8	27.9
5	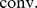 †	clusters	yes	11	547.5	41.6
6	conv.	clusters	no	10	622.5	116.6
7	conv.	yearly	yes	11	641.5	135.6
8	conv.	yearly	no	10	642.9	137.0

* Mixing based on physical contacts from POLYMOD survey [13].

† Mixing based on conversational contacts from POLYMOD survey [13].

**Figure 2:**
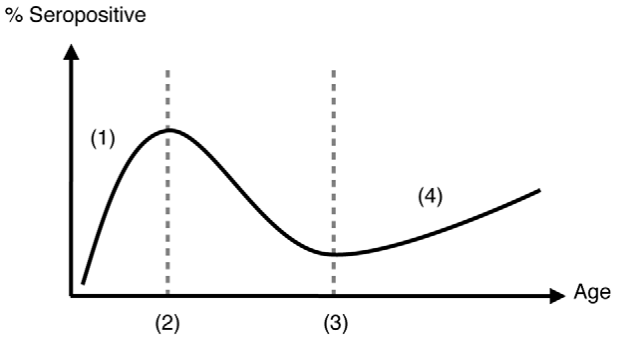
Schematic diagram of proposed mechanism behind the age profile of immunity. Individuals go through four different states as they age: 1) little prior immunity, so seropositivity increases with age and infection, as in a Poisson process; 2) hosts have memory B cells from previous exposures, so novel antibodies to circulating strain are less likely to be made and immunity drops as the strain evolves; 3) the virus has evolved out of the ‘reach’ of OAS, enabling new antibodies to be generated; 4) freedom from OAS, along with vaccination in the elderly, leads to an increase in seropositivity.

The decrease in seropositivity between the second and third age cohort is present in all datasets except Figure 1E. We propose that this is caused by the changing influence of OAS with age, as shown schematically in Figure 2. In the youngest age groups immunity gathers with infection: each exposure leads to an increased antibody repertoire. At the population level, this causes an increase in seropositivity with age. However, once a large proportion of individuals have been infected with at least one strain, OAS starts to have an effect, with subsequent infections re-stimulating existing antibodies rather than novel ones. The virus is still evolving, however, so the effective immunity to a new strain decreases with age until the virus is sufficiently different for the immune response to escape the effect of OAS, enabling an increase in immunity in older age groups. The maximum likelihood point estimate for the reach of OAS, 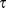, was 0.93. This implies that OAS can occur even if there is only a small degree of cross-reaction between strains, and that the preferential utilization of childhood immunity continues well into adult life. Based on our estimates for 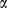 and 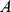 (Table S2), and assuming cluster change every 4 years on average for H1N1 and 3 years for H3N2 (Table S1), the temporal reach of OAS in the model ranges from 22–47 years.

For individuals who have seen at least two strains, the parameter estimates from model 1 suggest that, on average, the time between seeing the first and second strain is 6.0 years (details in Text S1). The overall average time between infections for each age class is given in Figure S2, with children seeing infection more often than older age groups.

**Figure 3:**
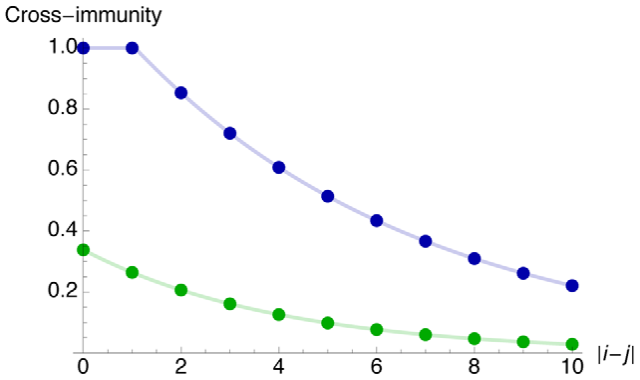
Estimated cross-immunity between clusters of strains. For two clusters of strains 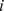 and 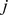, the level of cross-immunity is given by 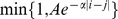. Green line, H1N1; blue line, H3N2.

Figure 3 shows the estimated degree of cross-immunity between clusters of strains, based on estimates in Table S2. Both subtypes have a similar decay over time, but the best fitting model suggests that H3N2 generates a noticeably higher degree of cross-immunity than H1N1; this parameter fit is a result of the large proportion of individuals seropositive to H3N2 in Finland (Figure 1D).

The estimates for 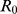 and 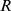 are shown in Table 3, with 95% confidence intervals in brackets (see Text S1 for details); these are slightly lower than previous estimates for 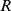, based on observed epidemic data [34], [35]. Note that data from Norway and France are shown for comparison, as published estimates for Finland could not be found.

**Table 3:** Estimated 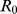 and 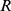 for different regions and subtypes.

Country	Subtype	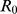 estimate	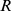 estimate	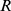 in empirical studies
Finland	H1N1	1.24 (1.19–1.30)	1.06 (1.03–1.22)	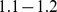 *
	H3N2	2.15 (2.03–2.28)	1.04 (0.54–2.06)	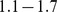 *
	Either			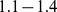 †
Australia	H1N1	1.40 (1.33–1.50)	1.15 (1.08–1.37)	
	H3N2	1.11 (1.09–1.13)	1.00 (0.91–1.02)	
	Either			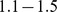 ‡

Model estimates of expected value are shown, with confidence interval in parentheses. Values of 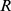 calculated in empirical studies are provided for comparison. ‘Either’ means that subtype was not specified.

* Data from Norway [35].

† Data from France [34].

‡ Data from Australia [34].

## Discussion

Using a multiple strain epidemic model, we have examined the age profile of immunity seen in serological data for seasonal influenza A in humans. Of the models considered, we have shown that the patterns observed in these studies can be best explained with a model which includes transmission based on physical contacts, antigenic clusters and original antigenic sin.

Although the model reproduces the general shape of much of the data, it does not fit as well to the Australian data for H3N2/Brisbane/07 (Figure 1F): there is a substantial drop in immunity after childhood that is not captured. This could be owing to a combination of factors. Some of these would be inconsistent with the other data, such as a higher rate of evolution (i.e. larger 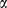) in Australia than in Finland. Others, such as a specific feedback over time between 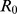 and virus evolution, would require numerous extra parameters, reducing the parsimony of the model. There may also be a discrepancy between the proportion of individuals with HI titre >40 and the true level of population immunity [16], [17], whereas we have assumed that individuals who are seropositive do not transmit infection. Microneutralisation assays provide a more sensitive and more specific method of measuring immune response than HI assays [36], however a correlate of protection has not yet been established for such tests [37]. The relationship may also be further complicated if in reality immunity offers clinical, but not transmission-blocking protection. A discrepancy between seropositivity and immunity may explain the difference in level of cross-protection between H1N1 and H3N2 strains in Figure 3.

Our results, which reproduce the decline in seropositivity between adolescence and middle age, are consistent with OAS occurring in combination with antigenic change in the virus. The effect is illustrated by level of immunity to H1N1/New Caledonia/99 (Figure 1B). Vaccine updates [25], [26] indicate that this strain was in a cluster that circulated until around 2006. However, in 2009 school children had the highest level of immunity to this virus, even though young adults would have been in the high-transmission school environment when this strain originally appeared in 1999. The first infection of a young adult would likely have been with a pre-1999 strain, though, so original antigenic sin could have subsequently inhibited the creation of specific immunity against New Caledonia/99 during these individuals' school years. This would not have been an issue for children born post-1999.

The maximum likelihood estimate for the reach of OAS, 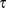, in the best-fitting model was 0.93. This suggests that childhood immunity dominates even if there is only a small degree of cross-immunity between strains. If antigenic evolution occurs at the rate suggested by the decay in Figure 3, this would mean that OAS still influences immunity several decades into an individual's life. OAS is likely caused by competition between existing memory B cells and naive ones for antigen [38], [39]. The estimate for 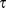 therefore suggests that even if persisting antibodies to the first strain seen have limited effectiveness against the epitopes of the current virus, they still prevent the activation of naive B cells, which would produce more effective antibodies [39]. If antigenic sin can recur, with memory B cells formed later in life also outcompeting naive ones, then a smaller reach would be required to generate a drop in immunity in middle age groups [14]. The increase in immunity in the elderly (Figure 2) would no longer be observed either; as subsequent strains could also induce antigenic sin, there would no longer be an age at which individuals could ‘escape’ its effects. Further studies into OAS could help address this issue. By examining the within-host interaction between B cells that likely generates antigenic sin, it would become clearer whether our simple version of OAS is sufficient in models of population immunity, or if a more detailed set of assumptions – such as the recently proposed ‘antigenic seniority’ hypothesis [40] – is required.

If antigenic sin could recur, it would also have implications for the inference of vaccination effectiveness, 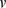. Using model 1, which assumes OAS, we estimated 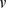 to be 6% for H1N1 and 24% for H3N2. A meta-analysis of empirical work into vaccine effectiveness [41] suggested vaccination reduced ILI by 35% (95% confidence interval 19–47%), and a review of vaccine efficacy [42] suggested a value of 17–53% for clinical efficacy in the elderly. Our estimates are low compared to these values. However, in our model 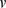 is defined as the additional reduction of transmission provided by vaccination, so its inferred value depends on the level of existing immunity in the elderly; if antigenic sin could recur, then the elderly would gain less natural immunity [14], and so we would expect the fitted value of 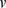 to increase.

Vaccination is implemented by removing a fixed proportion of the elderly from the 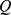 compartment at the start of each season. Cross-protection can therefore be considered by interpreting 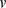 as the protection conferred from that season's vaccine as well as cross-immunity from previous years' campaigns. As a result, we would not expect the introduction of explicitly cross-protective vaccine (i.e. one that also removed individuals from future 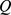 compartments) to substantially change our results, other than the estimate for 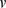. Interestingly, an increase in seroprevalence in older age groups has also been observed in populations with few vaccinated individuals [43], which suggests it may not be vaccination alone that is responsible for the rise in the elderly outlined in Figure 2.

The estimates for the effective reproductive ratio, 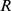, given in Table 3 are lower than estimates obtained in epidemiological studies. However, it is worth noting that the real-life estimates for France and Australia [34] did not include mild epidemics, whereas here 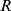 is estimated from values of 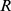 calculated for each year. As such, confidence intervals for 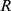 can extend below one if epidemics do not occur in every year of the simulations, as happens for H3N2. The estimates in Table 3 also suggest that there may not always be an easily discernible relationship between 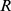, which can be estimated from data [34], [35], and 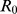, which often cannot. H3N2 in Finland has the largest fitted 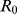, yet a high transmission rate results in a large degree of immunity, reducing 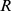.

Several assumptions have been made in the model presented here. We assume that immunity reduces transmission, rather than susceptibility: antibodies from past exposures do not prevent an individual from acquiring new infections – and adding these strains to their infection history – but they may prevent that person from transmitting the infection to others. Whereas a simple mechanism is used to represent OAS in the model, in reality factors such as cross-reaction, OAS and 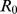 are likely to be subject to additional variation – both within-host [38] and at the environment level [44] – that is not included in the framework we have outlined here. In addition, transmission rates between age groups were based on age mixing data from the POLYMOD survey [13]. It has previously been shown that a model based on physical contacts from POLYMOD fits well to serological data for varicella and parvovirus [11], and our results suggest the same for influenza: it appears that transmission based on physical contacts captures influenza dynamics better than that based on conversational interactions. When conversational contact data is used in the model, much transmission occurs amongst the eldest two age classes; with physical contact data this bias is smaller, with more transmission to and from the younger classes. Further research into the role of school children [12] – who display a large amount of immunity relative to other age groups – in transmission might provide the detail required to better understand why physical contacts appear to be more relevant than conversational ones. Finally, we have only fitted the model to data from two studies: if more data on seropositivity to seasonal strains were to become available it might help elucidate some of the above issues. It may also improve our understanding of seasonal epidemics; although the effect of the immune structure of a population on an outbreak is well documented for pandemics [37], [45], it has been suggested that previous infections can have counter-intuitive implications for epidemic dynamics, with OAS leading to ‘blind spots’ in immunity [14].

To our knowledge, this is the first time a high-dimensional model of disease strains has been quantitatively compared with serological data for seasonal influenza. As well as highlighting the role played by age-dependent mixing and original antigenic sin, our work suggests that there may be an additional mechanism involved in shaping population immunity still to be identified. Further empirical studies into the immune structure of a population, interfaced with strain models such as the one presented here, are therefore essential if we are to fully understand how individuals build immunity to diseases like influenza.

## Supporting Information

Figure S1
**Distribution of population with age.** Red, data from Finland [29]; blue, data from Australia [28]; black, 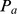 in model.(TIFF)

Figure S2 **Estimated average time between infections for each country and subtype.** Calculated as the median of the values in Table S3 for H1N1 and H3N2 in Finland, and H1N1 in Australia.
(TIFF)

Figure S3
**Convergence plots for the four** 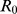 **parameters in model 1.** The black lines give the maximum likelihood point estimate, 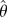.(TIFF)

Figure S4
**Sliced likelihood plots for the four** 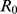 **parameters in model 1.** Points represent 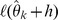, with the solid line at 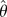 and confidence intervals given by dashed lines.(TIFF)

Table S1 **Assumed dates of appearance of new clusters.**
(PDF)

Table S2 **Parameter estimates obtained in the eight models.**
(PDF)

Table S3 **Estimated average time between infections in years, by age class.**
(PDF)

Text S1 **Provides details of model derivation, discussion of estimates for time between infections, and diagnostic tests for the inference framework.**
(PDF)

## References

[1] Wilson I, Cox N (1990) Structural basis of immune recognition of influenza virus hemagglutinin. Annu Rev Immunol 8: 737–787. 2188678 10.1146/annurev.iy.08.040190.003513

[2] Gilbert GL, Cretikos MA, Hueston L, Doukas G, O'Toole B, et al (2010) Influenza a (h1n1) 2009 antibodies in residents of new south wales, australia, after the first pandemic wave in the 2009 southern hemisphere winter. PLoS One 5: e12562. 20830210 10.1371/journal.pone.0012562 PMC2935357

[3] Ikonen N, et al (2010) High frequency of cross-reacting antibodies against 2009 pandemic influenza a(h1n1) virus among the elderly in finland. Eurosurveillance 15. 20144443

[4] Johnson BF, Wilson LE, Ellis J, Elliot AJ, Barclay WS, et al (2009) Fatal cases of influenza a in childhood. PLoS One 4: e7671. 19876396 10.1371/journal.pone.0007671 PMC2764845

[5] Mak GC, Choy PWW, Lee WY, Wong AH, Ng KC, et al (2010) Sero-immunity and serologic response to pandemic influenza a (h1n1) 2009 virus in hong kong. J Med Virol 82: 1809–15. 20872705 10.1002/jmv.21895

[6] Skowronski DM, Hottes TS, McElhaney JE, Janjua NZ, Sabaiduc S, et al (2011) Immunoepidemiologic correlates of pandemic h1n1 surveillance observations: higher antibody and lower cell-mediated immune responses with advanced age. J Infect Dis 203: 158–67. 21288814 10.1093/infdis/jiq039 PMC3071066

[7] Tandale B, Pawar S, Gurav Y, Chadha M, Koratkar S, et al (2010) Seroepidemiology of pandemic influenza A(H1N1) 2009 virus infections in pune, india. BMC Infect Dis 10: 255. 20738878 10.1186/1471-2334-10-255 PMC2936412

[8] Zimmer SM, Crevar CJ, Carter DM, Stark JH, Giles BM, et al (2010) Seroprevalence following the second wave of pandemic 2009 h1n1 influenza in pittsburgh, pa, usa. PLoS One 5: e11601. 20644650 10.1371/journal.pone.0011601 PMC2904390

[9] Grenfell B, Anderson R (1985) The estimation of age-related rates of infection from case notifications and serological data. Epidemiol Infect 95: 419–436. 10.1017/s0022172400062859 PMC2129533 4067297

[10] Anderson R, May R (1985) Age-related changes in the rate of disease transmission: implications for the design of vaccination programmes. J Hyg (Camb) 94: 365–436. 4008922 10.1017/s002217240006160x PMC2129492

[11] Melegaro A, Jit M, Gay N, Zagheni E, Edmunds WJ (2011) What types of contacts are important for the spread of infections?: using contact survey data to explore European mixing patterns. Epidemics 3: 143–51. 22094337 10.1016/j.epidem.2011.04.001

[12] Conlan AJK, Eames KTD, Gage JA, von Kirchbach JC, Ross JV, et al (2011) Measuring social networks in british primary schools through scientific engagement. Proc Biol Sci 278: 1467–75. 21047859 10.1098/rspb.2010.1807 PMC3081745

[13] Mossong J, Hens N, Jit M, Beutels P, Auranen K, et al (2008) Social contacts and mixing patterns relevant to the spread of infectious diseases. PLoS Med 5: e74. 18366252 10.1371/journal.pmed.0050074 PMC2270306

[14] Kucharski AJ, Gog JR (2012) Age profile of immunity to influenza: effect of original antigenic sin. Theor Popul Biol 81: 102–112. 22209755 10.1016/j.tpb.2011.12.006

[15] Francis T Jr (1960) On the doctrine of original antigenic sin. Proc Am Philos Soc 104: 572–578.

[16] Hobson D, Curry R, Beare A, Ward-Gardner A (1972) The role of serum haemagglutination-inhibiting antibody in protection against challenge infection with influenza a2 and b viruses. J Hyg (Lond) 70: 767–777. 4509641 10.1017/s0022172400022610 PMC2130285

[17] Potter CW (1979) Determinants of immunity to influenza infection in man. Br Med Bull 35: 69–75. 367490 10.1093/oxfordjournals.bmb.a071545

[18] Andreasen V (2003) Dynamics of annual influenza a epidemics with immuno-selection. J Math Biol 46: 504–536. 12783180 10.1007/s00285-002-0186-2

[19] Boni MF (2008) Vaccination and antigenic drift in influenza. Vaccine 26S: C8–C14. 10.1016/j.vaccine.2008.04.011 PMC2603026 18773534

[20] Rambaut A, Pybus OG, Nelson MI, Viboud C, Taubenberger JK, et al (2008) The genomic and epidemiological dynamics of human influenza a virus. Nature 453: 615–619. 18418375 10.1038/nature06945 PMC2441973

[21] Russell CA, Jones TC, Barr IG, Cox NJ, Garten RJ, et al (2008) The global circulation of seasonal influenza a (h3n2) viruses. Science 320: 340–46. 18420927 10.1126/science.1154137

[22] Lavenu A, Leruez-Ville M, Chaix M, Boelle P, Rogez S, et al (2005) Detailed analysis of the genetic evolution of influenza virus during the course of an epidemic. Epidemiol Infect 134: 514–520. 16316493 10.1017/S0950268805005686 PMC2870435

[23] Nelson MI, Edelman L, Spiro DJ, Boyne AR, Bera J, et al (2008) Molecular epidemiology of a/h3n2 and a/h1n1 influenza virus during a single epidemic season in the united states. PLoS Pathog 4: e1000133. 18725925 10.1371/journal.ppat.1000133 PMC2495036

[24] Smith DJ, Lapedes AS, de Jong JC, Besterbroer TM, Rimmelzwaan GF, et al (2004) Mapping the antigenic and genetic evolution of influenza virus. Science 305: 371–376. 15218094 10.1126/science.1097211

[25] Anker M, Schaaf D (2000) Who report on global surveillance of epidemic-prone infectious diseases. World Health Organisation

[26] World Health Organisation (2009) Recommended composition of influenza virus vaccines. Wkly Epidemiol Rec

[27] Andreasen V (2011) The final size of an epidemic and its relation to the basic reproduction number. Bull Math Biol 73: 2305–21. 21210241 10.1007/s11538-010-9623-3 PMC7088810

[28] Australian Bureau of Statistics (2010) Population by age and sex, australian states and territories. Australian Demographic Statistics (cat no 31010) Available from: http://abs.gov.au.

[29] Official Statistics of Finland (2010) Population structure [e-publication] ISSN = 1797- 5395. Helsinki: Statistics Finland Access method: http://www.stat.fi.

[30] Blank P, Schwenkglenks M, Szucs T (2009) Vaccination coverage rates in eleven european countries during two consecutive influenza seasons. J Infect 58: 446–458. 19446340 10.1016/j.jinf.2009.04.001

[31] Gill TK, Taylor AW, Watson M (2007) Trends in influenza immunisation amongst an elderly australian community. Vaccine 25: 5428–32. 17559991 10.1016/j.vaccine.2007.04.073

[32] Gilks W, Richardson S, Spiegelhalter D (1996) Markov chain Monte Carlo in practice. Boca Raton, FL: Chapman & Hall/CRC.

[33] Burnham K, Anderson D (2002) Model selection and multimodel inference: a practical information-theoretic approach. Second edition. New York: Springer-Verlag.

[34] Chowell G, Miller MA, Viboud C (2008) Seasonal influenza in the united states, france, and australia: transmission and prospects for control. Epidemiol Infect 136: 852–864. 17634159 10.1017/S0950268807009144 PMC2680121

[35] Gran JM, Iversen B, Hungnes O, Aalen OO (2010) Estimating influenza-related excess mortality and reproduction numbers for seasonal influenza in norway, 1975–2004. Epidemiol Infect 138: 1559–68. 20334732 10.1017/S0950268810000671

[36] Grund S, Adams O, Wählisch S, Schweiger B (2011) Comparison of hemagglutination inhibition assay, an elisa-based micro-neutralization assay and colorimetric microneutralization assay to detect antibody responses to vaccination against influenza a h1n1 2009 virus. J Virol Methods 171: 369–73. 21146560 10.1016/j.jviromet.2010.11.024

[37] Miller E, Hoschler K, Hardelid P, Stanford E, Andrews N, et al (2010) Incidence of 2009 pandemic influenza a h1n1 infection in england: a cross-sectional serological study. Lancet 375: 1100–8. 20096450 10.1016/S0140-6736(09)62126-7

[38] Kim J, Skountzou I, Compans R, Jacob J (2009) Original antigenic sin responses to influenza viruses. Journal Immunol 183: 3294. 19648276 10.4049/jimmunol.0900398 PMC4460008

[39] Lambert PH, Liu M, Siegrist CA (2005) Can successful vaccines teach us how to induce efficient protective immune responses? Nat Med 11: S54–62. 15812491 10.1038/nm1216

[40] Lessler J, Riley S, Read JM, Wang S, Zhu H, et al (2012) Evidence for antigenic seniority in influenza a (h3n2) antibody responses in southern china. PLoS Pathog 8: e1002802. 22829765 10.1371/journal.ppat.1002802 PMC3400560

[41] Vu T, Farish S, Jenkins M, Kelly H (2002) A meta-analysis of effectiveness of influenza vaccine in persons aged 65 years and over living in the community. Vaccine 20: 1831–6. 11906772 10.1016/s0264-410x(02)00041-5

[42] Goodwin K, Viboud C, Simonsen L (2006) Antibody response to influenza vaccination in the elderly: a quantitative review. Vaccine 24: 1159–69. 16213065 10.1016/j.vaccine.2005.08.105

[43] Lessler J, Cummings DAT, Read JM, Wang S, Zhu H, et al (2011) Location-specific patterns of exposure to recent pre-pandemic strains of influenza a in southern china. Nat Commun 2: 423. 21829185 10.1038/ncomms1432 PMC3757505

[44] Truscott J, Fraser C, Cauchemez S, Meeyai A, Hinsley W, et al (2011) Essential epidemiological mechanisms underpinning the transmission dynamics of seasonal influenza. J R Soc Interface 9: 304–312. 21715400 10.1098/rsif.2011.0309 PMC3243394

[45] Reichert T, Chowell G, Nishiura H, Christensen R, McCullers J (2010) Does glycosylation as a modifier of original antigenic sin explain the case age distribution and unusual toxicity in pandemic novel h1n1 influenza? BMC Infect Dis 10: 5. 20059763 10.1186/1471-2334-10-5 PMC3003248

