# Supplementary figures and images for: The Role of Social Contacts and Original Antigenic Sin in Shaping the Age Pattern of Immunity to Seasonal Influenza
Source: PLoS Comput Biol. 2012 Oct 25;8(10):e1002741. doi: 10.1371/journal.pcbi.1002741 (PMC3486889; doi:10.1371/journal.pcbi.1002741)

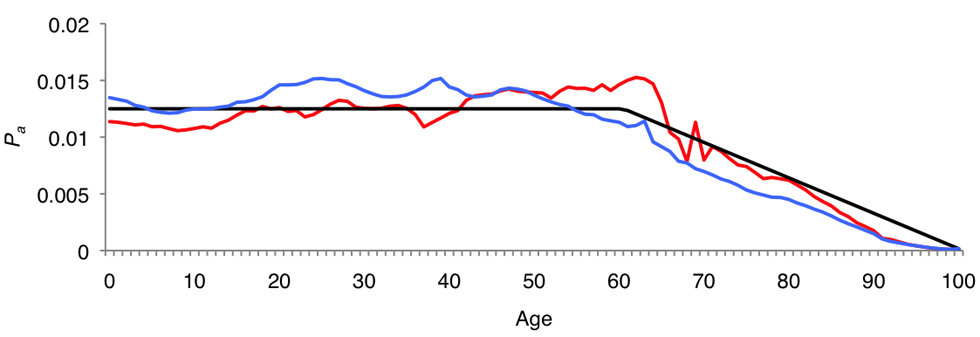

Supplement: Figure S1 — Distribution of population with age. Red, data from Finland [29]; blue, data from Australia [28]; black, in model. (TIFF) [file pcbi.1002741.s001.tiff]

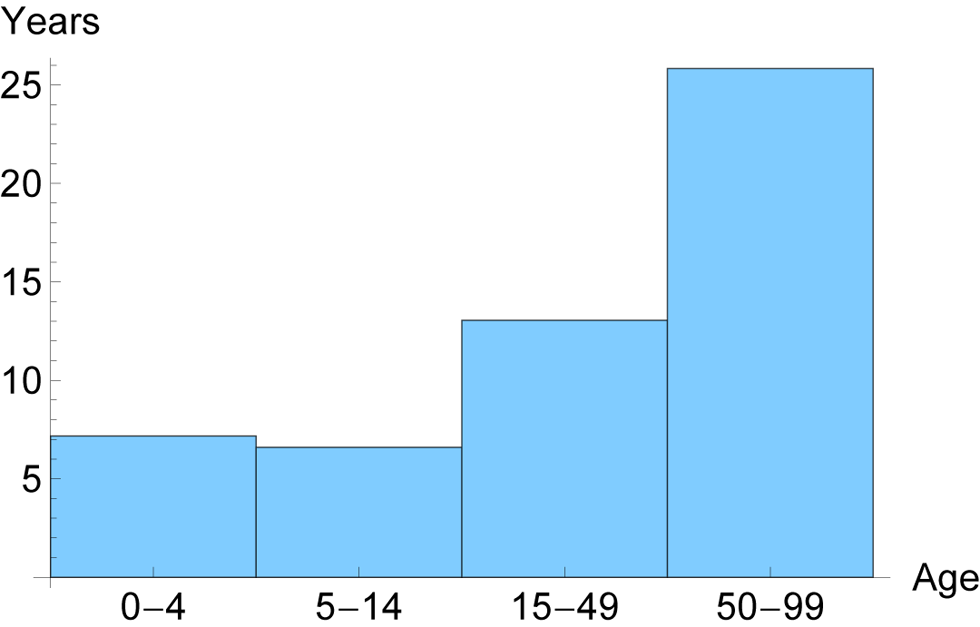

Supplement: Figure S2 — Estimated average time between infections for each country and subtype. Calculated as the median of the values in Table S3 for H1N1 and H3N2 in Finland, and H1N1 in Australia. (TIFF) [file pcbi.1002741.s002.tiff]

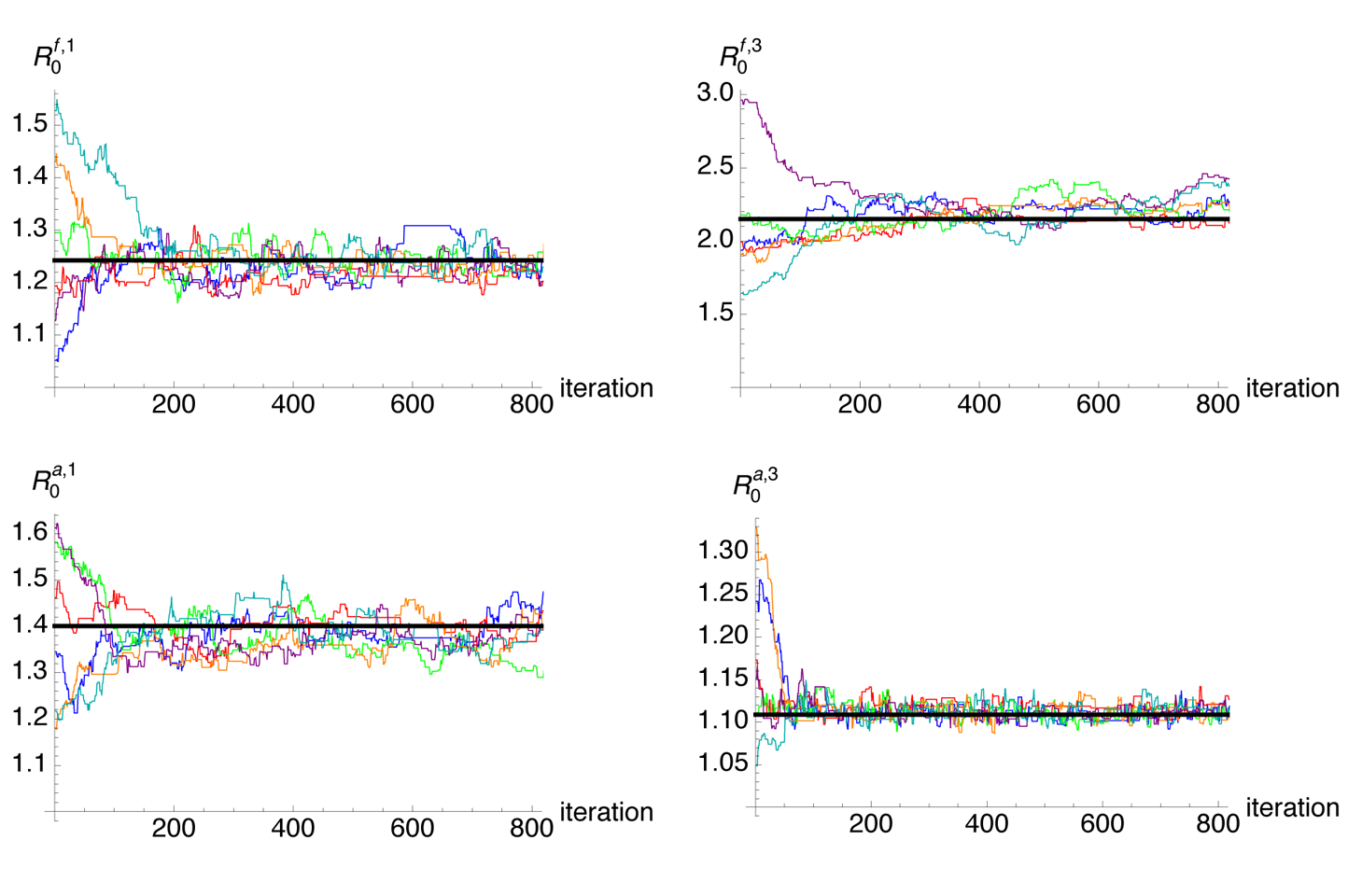

Supplement: Figure S3 — Convergence plots for the four parameters in model 1. The black lines give the maximum likelihood point estimate, . (TIFF) [file pcbi.1002741.s003.tiff]

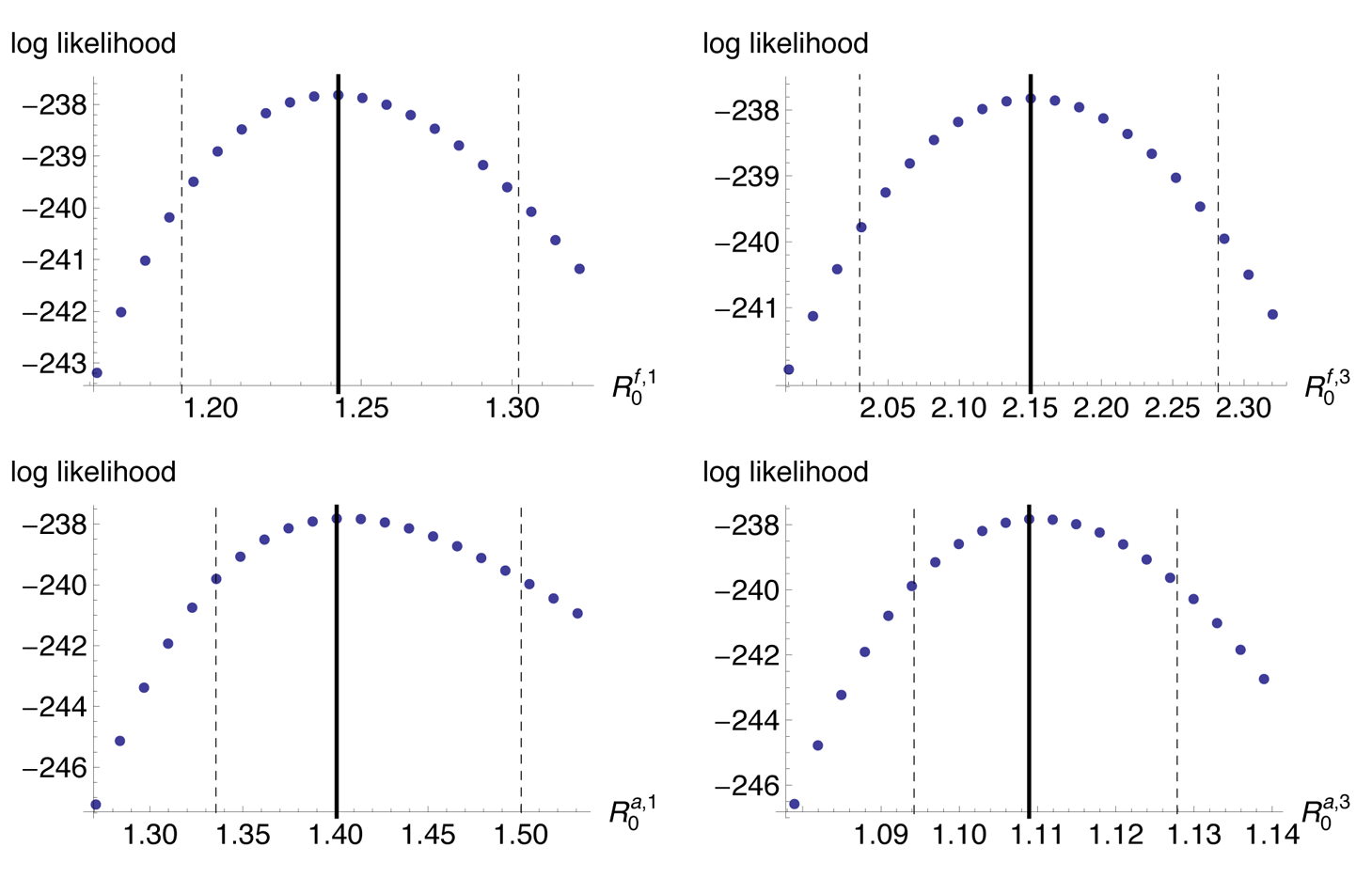

Supplement: Figure S4 — Sliced likelihood plots for the four parameters in model 1. Points represent , with the solid line at and confidence intervals given by dashed lines. (TIFF) [file pcbi.1002741.s004.tiff]
