# Supplementary material for: The Role of Social Contacts and Original Antigenic Sin in Shaping the Age Pattern of Immunity to Seasonal Influenza
Source: PLoS Comput Biol. 2012 Oct 25;8(10):e1002741. doi: 10.1371/journal.pcbi.1002741 (PMC3486889; doi:10.1371/journal.pcbi.1002741)
Supplement: Table S1 — Assumed dates of appearance of new clusters. (PDF) [file pcbi.1002741.s005.pdf]

Table S1: **Assumed dates of appearance of new clusters.**

Subtype	Year	Cluster
H1N1	1977	A/USSR/90/77
	1979	A/Brazil/11/78
	1983	A/Chile/1/83
	1986	A/Singapore/6/86
	1996	A/Bayern/7/95
	1997	A/Beijing/262/95
	1999	A/New Caledonia/20/99
	2006	A/Solomon Islands/3/2006
	2007	A/Brisbane/59/2007
	2009	A/California/7/2009 *
H3N2	1968	A/Hong Kong/1/68
	1972	A/England/42/72
	1976	A/Victoria/3/75
	1978	A/Texas/1/77
	1980	A/Bangkok/1/79
	1987	A/Shanghai/11/87
	1989	A/Beijing/353/89
	1992	A/Beijing/32/92
	1996	A/Wuhan/359/95
	1997	A/Sydney/5/97
	2003	A/Fujian/411/02
	2004	A/California/7/04
	2005	A/Wisconsin/67/05
	2007	A/Brisbane/07
	2009	A/Perth/09

*As the pandemic H1N1 strain did not arise from antigenic drift, we assume that no other strain gives immunity to this strain, and vice versa.
