# Supplementary material for: The Role of Social Contacts and Original Antigenic Sin in Shaping the Age Pattern of Immunity to Seasonal Influenza
Source: PLoS Comput Biol. 2012 Oct 25;8(10):e1002741. doi: 10.1371/journal.pcbi.1002741 (PMC3486889; doi:10.1371/journal.pcbi.1002741)
Supplement: Table S2 — Parameter estimates obtained in the eight models. (PDF) [file pcbi.1002741.s006.pdf]

Table S2: **Parameter estimates obtained in the eight models.**

Model	τ	$R_0^{f,1}$	$R_0^{f,3}$	$R_0^{a,1}$	$R_0^{a,3}$	α_1	α_3	A_1	A_3	v_1	v_3
1	0.93	1.24	2.15	1.40	1.11	0.25	0.17	0.34	1.20	0.06	0.24
2	–	1.22	2.02	1.27	1.11	1.22	0.85	0.35	1.71	0.13	0.23
3	0.20	1.23	2.17	1.36	1.10	0.15	0.34	0.27	1.07	0.10	0.21
4	–	1.26	2.25	1.31	1.10	0.19	0.32	0.31	0.95	0.10	0.21
5	0.96	1.23	2.10	1.45	1.13	0.24	0.16	0.29	1.09	0.07	0.16
6	–	1.32	5.13	1.83	1.12	0.76	0.86	0.15	0.55	0.07	0.17
7	0.40	1.32	3.35	1.69	1.10	0.12	0.27	0.19	0.65	0.06	0.16
8	–	1.33	3.02	1.65	1.10	0.12	0.33	0.20	0.68	0.07	0.21
