# Supplementary material for: The Role of Social Contacts and Original Antigenic Sin in Shaping the Age Pattern of Immunity to Seasonal Influenza
Source: PLoS Comput Biol. 2012 Oct 25;8(10):e1002741. doi: 10.1371/journal.pcbi.1002741 (PMC3486889; doi:10.1371/journal.pcbi.1002741)
Supplement: Table S3 — Estimated average time between infections in years, by age class. (PDF) [file pcbi.1002741.s007.pdf]

Table S3: **Estimated average time between infections in years, by age class.**

Country	Subtype	0–4	5–14	15–49	50–99
Finland	H1N1	8.4	6.6	15.7	37.1
	H3N2	7.2	7.2	13.0	25.8
Australia	H1N1	4.1	3.4	7.3	16.6
	H3N2	>50	>50	>50	>50
