# Supplementary material for: The Role of Social Contacts and Original Antigenic Sin in Shaping the Age Pattern of Immunity to Seasonal Influenza
Source: PLoS Comput Biol. 2012 Oct 25;8(10):e1002741. doi: 10.1371/journal.pcbi.1002741 (PMC3486889; doi:10.1371/journal.pcbi.1002741)
Supplement: Text S1 — Provides details of model derivation, discussion of estimates for time between infections, and diagnostic tests for the inference framework. (PDF) [file pcbi.1002741.s008.pdf]

Supporting Text S1

The Role of Social Contacts and Original Antigenic Sin in Shaping the Age Pattern of Immunity to Seasonal Influenza

Adam J. Kucharski[1*], Julia R. Gog[1]

[1]Department of Applied Mathematics and Theoretical Physics, University of Cambridge, Cambridge, United Kingdom

*E-mail: ak640@cam.ac.uk

1 Model derivation

1.1 Demography function

In Australia and Finland, the equilibrium age distribution of the population, P_a, is relatively flat up to age 60, then decreases to zero [1, 2]. We therefore assume that the population is divided into 99 discrete ages, one for each year (no one survives to age 100), where $\sum_{a=1}^{99} P_a = 1$. Figure S1 shows that a simple piecewise function,

$$P_a = \begin{cases} 1/80 & \text{if } 0 < a \leq 60 \\ 1/32(1/195 - a/100) & \text{if } 60 < a \leq 99 \end{cases}$$

gives a reasonable approximation to the actual age distributions of these populations.

1.2 Age structured model

Rather than implement a system with continuous transmission and evolution, we consider only the outcome of each yearly epidemic [3], and assume that only one influenza virus circulates each year and that the annual epidemics do not overlap.

We denote $\sigma(Y, i)$ as the probability an individual will transmit a challenge strain in cluster i, given existing immunity to a set of strains Y,

$$\sigma(Y, i) = \max\{0, \min_{j \in Y}\{1 - Ae^{-\alpha|i-j|}\}\} \quad (1)$$

where $0 < A \leq 1$. Equation 1 can be approximated [4] by writing σ in the form

$$\sigma(Y, i) = \frac{1}{E} \sum_{k=1}^{E} \prod_{j \in Y} \sigma_{j,i}^{k} \quad (2)$$

$$\text{where} \quad \sigma_{j,i}^{k} = \begin{cases} 1 & \text{if } k \leq [E(1 - Ae^{-\alpha|i-j|})] \\ 0 & \text{else} \end{cases} \quad (3)$$

and $[x]$ is x rounded to the nearest integer. In this paper, we set $E = 25$, and Table S1 gives the years in which we assume there is a change of antigenic cluster.

We implement these assumptions using an age structured multi-strain model [4], which we extend to allow for age-dependent mixing for transmission. Denote the set of all strains as $\mathcal{N} = \{1, ..., n\}$, where there are n strains in total, and S_X as the proportion of the population that have been infected by all strains in set $X \subseteq \mathcal{N}$, but not by any other strains.

We define $\beta = \beta_{ab}$ as the rate of transmission to individuals aged a from individuals aged b, and $I_i(a, t)$ denotes the number of individuals aged a infective with a specific strain $i \in \mathcal{N}$. We assume each season consists of a single epidemic caused by one strain, starting and finishing with almost no infection, and aging is implemented between seasons. Focusing now on strain i, define

$$Q(a) = \sum_{Y \subset \mathcal{N}} \sigma(Y, i)\ S_Y(a)$$

to be the *potential infectivity* of the population [3]. This denotes the proportion of the population who are aged a and would have the potential to transmit if they acquired infection. We therefore obtain the following set of ODEs for season i [4, 5],

$$\frac{dQ(a,t)}{dt} = -\ Q(a,t) \sum_b \beta_{ab} I(b,t)$$

$$\frac{dI(a,t)}{dt} = Q(a,t) \sum_b \beta_{ab} I(b,t) - \gamma I(a,t)$$

Without loss of generality we set $\gamma = 1$ [5], and find the relative value of Q at the end of each epidemic, $\phi_a = Q(a, \infty)/Q(a, 0)$, by solving

$$F_a(\phi) = \sum_b Q(b, 0)\beta_{ab}(1 - \phi_b) + \log \phi_a = 0$$

numerically. If $\hat{S}_j(a)$ is the proportion of individuals who are aged a and have been infected in season i, then at the end of the epidemic, $\hat{S}_i(a) = (1 - \phi_a)P_a$.

Birth, death and ageing processes are implemented after each epidemic. This means individuals aged 99 are removed; those between 1 to 98 are moved into the next age class; and P_1 newborns are added. $\hat{S}_i$ must also be adjusted to account for deaths as age increases: this is achieved by setting

$$\hat{S}_i(a) = \frac{P_a}{P_{a-1}} \hat{S}_i(a-1)\ .$$

1.3 Construction of transmission matrices

For the four age classes we define m_{ij} as the average number of contacts in age class i recorded per day per survey participant in age class j. If $\{i_1, i_2\}$ denote the age boundaries of age class i, then the proportion of individuals in age class i is given by

$$P_i = \sum_{a=i_1}^{i_2} P_a$$

and hence transmission rate to age class i from j is

$$\beta_{ij} = km_{ij}/P_i$$

where k is some scaling factor dependent on the infection's basic reproductive ratio, R_0 [6]. P_i is included in the denominator as age classes are not equal size. As R_0 is equivalent to the maximal eigenvalue of the 4×4 matrix with entries

$$\begin{aligned} R_{i,j} &= \beta_{ij} P_i/\gamma \\ &= km_{ij}/\gamma \, , \end{aligned}$$

for a given R_0, we can also find numerically the value of k needed to scale β_{ij} correctly.

Age-dependent contacts, m_{ij}, are taken from the European POLYMOD survey in Finland [7], which includes data on both physical and non-physical contacts. A physical contact is defined as "skin-to-skin contact such as a kiss or handshake", and non-physical contact is defined as "a two-way conversation with three or more words in the physical presence of another person" [7]. In this paper, we define conversational contacts as all reported contacts (physical and non-physical).

2 Time between infections

2.1 Average time for each age class

The attack rate for age class a each season is given by $1 - \phi_a$, where ϕ_a is as defined in Section 1.2. For each age class, define ψ_a be the mean of these values over all seasons. The expected time between infections for each age class can therefore be estimated by calculating $1/\psi_a$. These values are given for each the country and subtype in Table S3. The high estimates

for time between infections (longer than the length of the simulation for all age classes) for H3N2 in Australia are the result of low R in each season (Table 3); in the inference model, these low attack rates appear necessary to compensate for the large cross-immunity estimate for H3N2 (Figure 3). As model 1 also gives a relatively poor fit to H3N2 in Australia (Figure 1), we therefore focus on the other three sets of estimates for time between infection. The median of these estimates is shown in Figure S2, with the average time between infections much lower for the high mixing 5–14 age class (6.6 years) than for the 50–99 age class (25.8 years).

2.2 Time from original to second infection

We can also calculate the overall average time between first and second infections using the mean attack rates. Let ψ_a now denote the mean attack rate for age group a (where $1 \leq a \leq 99$), and suppose a maximum of n strains could have been seen. Define T_y as the proportion of individuals who saw their second strain y years after their first, where

$$T_y = \sum_{i=1}^{n-y} \left[\prod_{j=1}^{i-1} (1 - \psi_j)\, \psi_i \prod_{j=i+1}^{i+y-1} (1 - \psi_j)\, \psi_{i+y} \right] .$$

The mean number of years from original to second infection (conditional on having seen at least two strains), T, is therefore

$$T = \frac{\sum_{y=1}^{n} T_y y}{\sum_{y=1}^{n} T_y} .$$

Using the values for ψ calculated in the previous section, we obtain estimates of 6.0 and 7.3 years for H1N1 and H3N2 in Finland, and 3.7 for H1N1 in Australia. The median of the three values is 6.0 (as in the previous section, we do not include the estimate for H3N2 in Australia, which was 13.1).

3 Diagnostics

3.1 Convergence plots

To check that the parameter estimate obtained in each model, $\hat{\theta}$, successfully converges to the maximum likelihood estimate for the model to the observed serology data, we use two diagnostic tests. The first is a convergence plot. Figure S3 shows the value of the four estimates of R_0 in model 1 at each iteration of the Metropolis-Hastings algorithm, starting with five different

initial parameter sets. These were generated from a multivariate normal distribution with mean $\hat{\theta}$ and covariance matrix $0.01I$, where I is the identity matrix. All converge to a similar parameter distribution, indicating that the method is robust to a perturbation of the initial value of θ. Similar convergence results were obtained for the other seven models.

3.2 Sliced likelihood and confidence intervals for R_0

The second diagnostic test is a 'sliced likelihood' [8]. Given the inferred parameter set, $\hat{\theta}$, the parameter in position k, $\hat{\theta}_k$, is varied by an amount h. The resulting $\ell(\hat{\theta}_k + h)$ is then plotted against $\hat{\theta}_k+h$. If $\hat{\theta}$ is positioned at the local maximum of each of the sliced likelihoods, it implies that $\hat{\theta}$ is a local maximum of $\ell(\theta)$. Figure S4 gives sliced likelihood for the four estimates of R_0 in model 1; in all cases, $\hat{\theta}$ is located at the maximum, suggesting that the likelihood has been successfully maximized. Similar convergence was observed in the other models. The 95.4% confidence interval is equivalent to a decrease of 2 in log-likelihood from the maximum [9]; these intervals are given by dashed lines in Figure S4.

3.3 Confidence intervals for R

The effective reproductive ratio for each season, R, is equivalent to the maximal eigenvalue of the 4×4 matrix with entries

$$\begin{aligned} r_{i,j} &= \beta_{ij} q_i / \gamma \\ &= k m_{ij} / \gamma \end{aligned}$$

where

$$q_i = \sum_{a=i_1}^{i_2} Q(a)$$

and $\{i_1, i_2\}$ denote the age boundaries of age class i.

To calculate the expected value of R, and its confidence intervals, we run the model 1000 times for each region and subtype, sampling R_0 from the converged distribution (as in Figure S3) on each run. We then take the simulated values of R calculated in each year of these runs (i.e. $1000y$ values in total, where y is the number of years simulated in each run), and find the median, as well as the upper and lower bound for the interval in which 95% of the values are

contained. The median gives an estimate of the expected value of R, and the upper and lower bound its confidence intervals.

References

[1] Australian Bureau of Statistics (2010) Population by age and sex, australian states and territories. Australian Demographic Statistics (cat no 31010) Available from: http://abs.gov.au.

[2] Official Statistics of Finland (2010) Population structure [e-publication] ISSN=1797-5395. Helsinki: Statistics Finland Access method: http://www.stat.fi.

[3] Andreasen V (2003) Dynamics of annual influenza a epidemics with immuno-selection. J Math Biol 46: 504-536.

[4] Kucharski AJ, Gog JR (2012) Age profile of immunity to influenza: effect of original antigenic sin. Theoretical Population Biology 81: 102-112.

[5] Andreasen V (2011) The final size of an epidemic and its relation to the basic reproduction number. Bull Math Biol .

[6] Wallinga J, Teunis P, Kretzschmar M (2006) Using data on social contacts to estimate age-specific transmission parameters for respiratory-spread infectious agents. American Journal of Epidemiology 164: 936.

[7] Mossong J, Hens N, Jit M, Beutels P, Auranen K, et al. (2008) Social contacts and mixing patterns relevant to the spread of infectious diseases. PLoS Med 5: e74.

[8] Ionides E, Breto C, King A (2006) Inference for nonlinear dynamical systems. Proceedings of the National Academy of Sciences 103: 18438.

[9] Hudson D (1971) Interval estimation from the likelihood function. Journal of the Royal Statistical Society Series B (Methodological) : 256–262.
